# Prognostic Significance of Neutrophil-to-Lymphocyte Ratio and C-Reactive Protein/Albumin Ratio in Luminal Breast Cancers With HER2-Negativity

**DOI:** 10.3389/fonc.2022.845935

**Published:** 2022-03-04

**Authors:** Fei Chen, Danzhi Chen, Lidan Jin, Chenpu Xu, Wenhe Zhao, Wenxian Hu

**Affiliations:** Department of Surgical Oncology, Sir Run Run Shaw Hospital, Zhejiang University School of Medicine, Hangzhou, China

**Keywords:** neutrophil-to-lymphocyte ratio (NLR), C-reactive protein/albumin ratio (CAR), breast cancer, disease-free survival (DFS), cancer-specific survival (CSS)

## Abstract

**Purpose:**

This study was determined to evaluate the prognostic value of neutrophil-to-lymphocyte ratio (NLR) and C-reactive protein/albumin ratio (CAR) prior to surgery in luminal breast cancers (BC) with HER2-negativity.

**Methods:**

The clinical data of 708 HER2-negative luminal BC patients from January 2013 to December 2016 were retrospectively collected and analyzed. The optimal cut-off value of NLR and CAR were determined *via* receiver operating characteristic (ROC) curve. The disease-free survival (DFS) and cancer specific survival (CSS) rates were estimated using the Kaplan−Meier method. Cox univariate and multivariate proportional hazards regression models were performed to identify significant predictors of DFS and CSS simultaneously.

**Results:**

The mean age of the patients diagnosed was 52.43 years (range, 15–95 years), and the median follow-up was 62.71 months (range, 12-92 months). Univariate and multivariate analysis confirmed that NLR ≥2.2 was significantly associated with worse DFS (HR=2.886, 95%CI=1.756-4.745, p<0.001), and same results were obtained in terms of CSS (HR=3.999, 95%CI=2.002-7.987, p<0.001). Similarly, CAR ≥0.07 was independently and significantly associated with poor DFS (HR=3.858, 95%CI=2.346-6.345, p<0.001) and CSS (HR=6.563, 95%CI=3.558-12.106, p<0.001).

**Conclusion:**

Preoperative evaluation of NLR and CAR were significant and independent prognostic indicators for luminal breast cancers with HER2-negativity.

## Introduction

Breast cancer (BC) is the most commonly diagnosed malignancy in women worldwide (1), with an estimated 20.9 million new cases and 626,679 deaths in 2018 (2). In China, the incidence and the mortality of BC account for 12.2% and 9.6% of the world respectively, and the growing trend has posed a serious threat to the health of women (3). Therefore, it is necessary to find a cheap, accurate and easily-available way to assess the prognosis of BC patients.

The systemic inflammatory state of the host and the inflammatory response in the tumor microenvironment are closely related to the proliferation, invasion and metastasis of tumor cells (4, 5). In recent years, inflammatory parameters such as neutrophil-to-lymphocyte ratio (NLR), platelet−to-lymphocyte ratio (PLR), lymphocyte-to-monocyte ratio (LMR), C-reactive protein/albumin ratio (CAR) and Glasgow Prognostic Score (GPS) have been reported as useful indicators for predicting the prognosis of various solid cancers, including BC (6–10). At present, the study concerning the correlation between NLR and the outcome of BC has become a research hotspot, most studies have shown that the elevation of NLR in peripheral blood is associated with worse prognosis in BC. In the most recent meta-analyses (11, 12), high NLR predicted poorer OS and DFS in BC patients, especially in triple-negative breast cancer (TNBC) patients. Controversial results were obtained when the predictive value of NLR was studied among HER-2+ BC patients administered with trastuzumab. Tiainen et al. (13) found that among the HER-2+ patients without trastuzumab therapy as adjuvant treatment, the survival rates of patients with high NLR were significantly lower compared with those with low NLR. On the contrary, Ding et al. (14) found that NLR did not have prognostic value among HER-2+ patients without trastuzumab treatment, yet could predict the prognosis of those who received trastuzumab treatment for 1 year. Additionally, the 3-year DFS of the low NLR group was also found to be significantly higher than that of the high NLR group (95.3% vs 90.5%, P=0.011). The majority of studies focused on the correlation between NLR and HER2-positive BC, whereas only a minority of studies investigated the possible predictive role of NLR in luminal BC with HER2-negativity (15, 16).

Recently, CAR has been reported to be an independent risk factor in several cancers, including esophageal cancer (17), gastric cancer (18), pancreatic cancer (19) and ovarian cancer (20) included, yet its prognostic role in BC remains to be elucidated. One study reported that an increased CAR before surgery was associated with a poor outcome in BC (9), yet another suggested that there was no correlation between CAR and BC outcome (21).

Taken together, this study is conducted to evaluate the prognostic value of NLR and CAR on DFS and CSS for luminal BC patients with HER2-negativity.

## Materials and Methods

### Patient Enrollment

A total of 708 breast cancer patients undergoing surgery between January 2013 and December 2016 at the Sir Run Run Shaw Hospital Affiliated to Zhejiang University School of Medicine were enrolled for inclusion in this retrospective study. All patients with a histopathologically confirmed BC and molecular pathologically confirmed luminal BC with HER-2 negative were included in the study. The exclusion criteria were as follow: 1) Patients with carcinoma in situ, terminal breast cancer on stage IV, inflammatory BC, and male sex; 2) Patients with other malignancies, inflammatory, autoimmune or hematological diseases, or in an immunosuppressed condition; 3) Patients without standard treatments based on the NCCN guidelines; 4) Patients without complete clinical data and death from non-tumor causes. This study was approved by the ethics committee of the Sir Run Run Shaw Hospital Affiliated to Zhejiang University School of Medicine according to the Declaration of Helsinki. There is no need for the informed consent of individual patients in written forms since this was a retrospective observational study.

### Pathological Characteristics

All histological data were reviewed by three pathologists. Hormonal status of Estrogen receptor (ER) and progesterone receptor (PR) was measured using Immunohistochemistry (IHC). Tumors with nuclear expression levels ≥ 1% were considered positive. HER-2 status was determined by IHC or fluorescent *in situ* hybridization (FISH). It was considered positive in the following situation: the IHC score was at least 3; or more than 2.2 times greater HER2 signals than CEP-17 signal in tumor cells was thought to be HER-2 amplification by FISH. The molecular types included in this study were ER positive and/or PR positive and HER2-negative, which could be further divided on the basis of Ki67 expression levels as follow: Luminal A, Ki67 < 14%, n=316; Luminal B, Ki67 ≥ 14%, n=392 (22).

### Laboratory Data

NLR and CAR levels within one week before treatment were retrieved from medical records. NLR was defined as neutrophil counts divided by lymphocyte counts. And neutrophil and lymphocyte counts were measured automatically using Mindray CAL8000 hematological analyzers (Mindray Corporation, Shenzhen, China). CAR was defined as the ratio of serum C-reactive protein (CRP) level divided by the serum albumin (Alb) level. Both CAR and Alb were measured automatically using Beckman Coulter AU5800 automatic biochemical analyzer (Beckman Coulter Corporation, California, USA).

### Follow-Up

Patients were followed-up regularly after the diagnosis of breast cancer. Follow-up was scheduled for every month in the first three months following the diagnosis, every six months for the first two years, and then every year. In this study, we set October 2020 as the deadline for follow-up. DFS was defined from the time of the diagnosis to the first event, including recurrence, metastasis and death. CSS was defined from the time of diagnosis to the death by cancer or the end of follow-up.

### Statistical Analysis

SPSS Statistics version 25.0 (IBM Corporation, Armonk, NY, USA) was used to perform statistical analysis. The ROC curve was established to determine the optimal cut-off value of NLR and CAR. The relationship between clinicopathological characteristics and NLR or CAR levels were calculated by Fisher’s exact test or chi-squared test. The DFS and CSS rates were estimated by the method of Kaplan−Meier analysis and differences in survival among different groups were compared using the Log-rank test. Uni- and multivariate prognostic analyses were performed to identify significant predictors of CSS and DFS using the Cox proportional hazards regression model. P-values < 0.05 were regarded as statistically significant.

## Results

### Patients Baseline Characteristics

**Figure 1:**
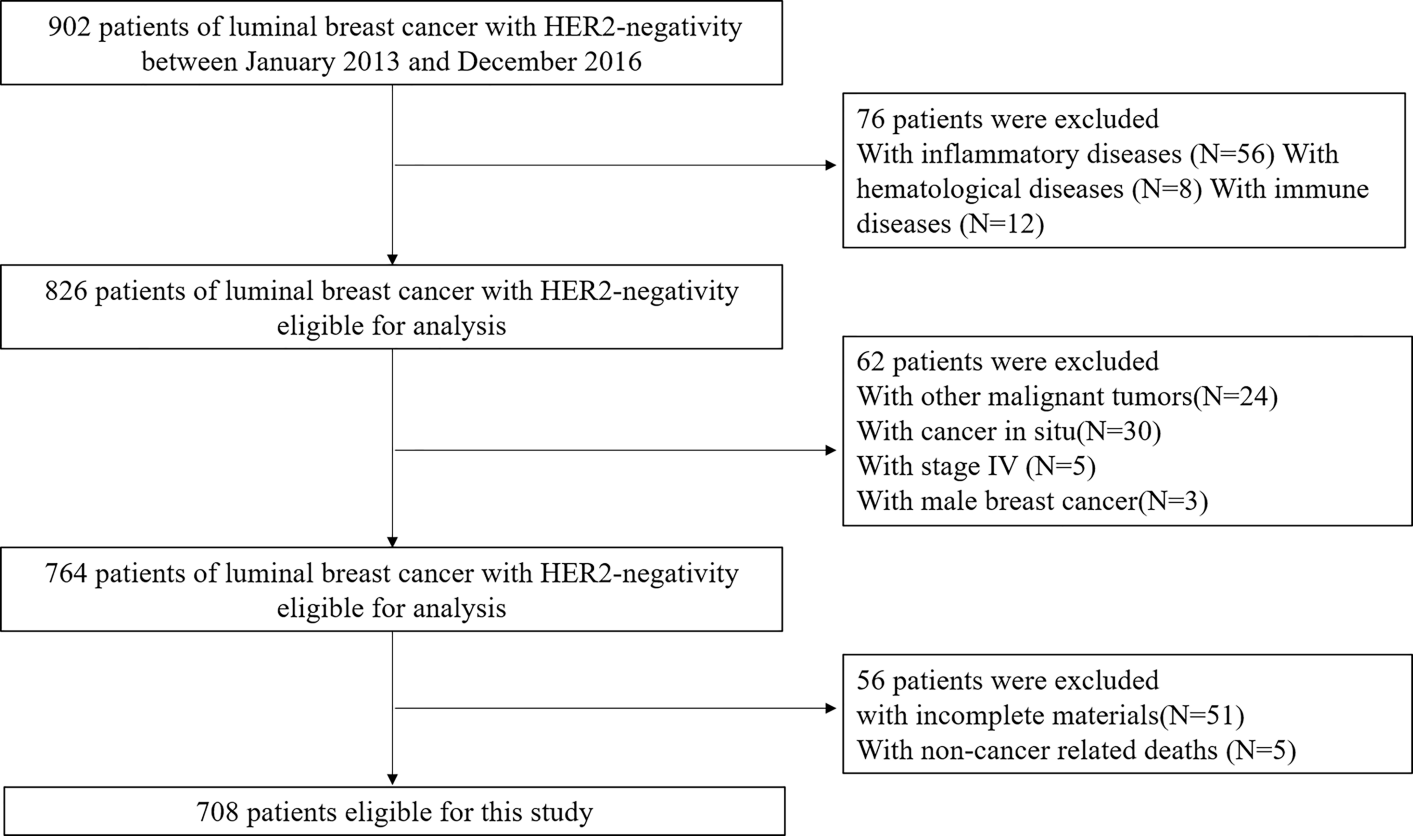
Study flow chart.

We identified 918 patients who completed the treatment of luminal BC with HER2-negativity, and 708 patients were eligible for analysis. The reasons for the excluded patients are summarized in **Figure 1**, and the baseline characteristics of included patients were displayed in **Table 1**. The mean age at diagnosis was 52.43 years (range, 15–95 years). The median follow-up was 62.71 months (range, 12-92 months). During follow-up, 77 patients suffered from progression of the disease, and 45 patients died. The DFS and CSS rates were 89.1% and 93.6%, respectively.

**Table 1:** Clinicopathologic Characteristics of 708 luminal breast cancers with HER2-negativity.

Characteristics	Number (%)
Age(years)	
<50	346 (48.9)
≥50	362 (51.1)
BMI	
<24	446 (63.0)
≥24	262 (37.0)
Menopausal status	
Pre-	362 (51.1)
Post-	327 (46.2)
Unknown	19 (2.7)
Tumor size	
<2cm	449 (63.4)
≥2cm	259 (36.6)
Lymph node metastasis	
Negative	607 (85.7)
Positive	101 (14.3)
TNM grade	
I	422 (59.6)
II	224 (31.6)
III	62 (8.8)
Histology type	
Infiltrating ductal carcinoma	635 (89.7)
Infiltrating lobular carcinoma	15 (2.1)
Others	57 (8.1)
Luminal subtype	
Luminal A	316 (44.6)
Luminal B	392 (55.4)
Operation type	
Modified radical mastectomy	375 (53.0)
Breast-conserving surgery	249 (35.2)
Mammectomy	72 (10.2)
Others	12 (1.7)
Chemotherapy	
Neoadjuvant chemotherapy	66 (9.3)
Adjuvant chemotherapy	362 (51.1)
No chemotherapy	280 (39.5)
Radiotherapy	
No	310 (43.8)
Yes	398 (56.2)
Characteristics	Number (%)
Age (years)	
<50	346 (48.9)
≥50	362 (51.1)
BMI	
<24	446 (63.0)
≥24	262 (37.0)
Menopausal status	
Pre-	362 (51.1)
Post-	327 (46.2)
Unknown	19 (2.7)
Tumor size	
<2cm	449 (63.4)
≥2cm	259 (36.6)
Lymph node metastasis	
Negative	607 (85.7)
Positive	101 (14.3)
TNM grade	
I	422 (59.6)
II	224 (31.6)
III	62 (8.8)
Histology type	
Infiltrating ductal carcinoma	635 (89.7)
Infiltrating lobular carcinoma	15 (2.1)
Others	57 (8.1)
Luminal subtype	
Luminal A	316 (44.6)
Luminal B	392 (55.4)
Operation type	
Modified radical mastectomy	375 (53.0)
Breast-conserving surgery	249 (35.2)
Mammectomy	72 (10.2)
Others	12 (1.7)
Chemotherapy	
Neoadjuvant chemotherapy	66 (9.3)
Adjuvant chemotherapy	362 (51.1)
No chemotherapy	280 (39.5)
	
Radiotherapy	
No	310 (43.8)
Yes	398 (56.2)

### The Cut-Off Values of NLR and CAR

**Figure 2:**
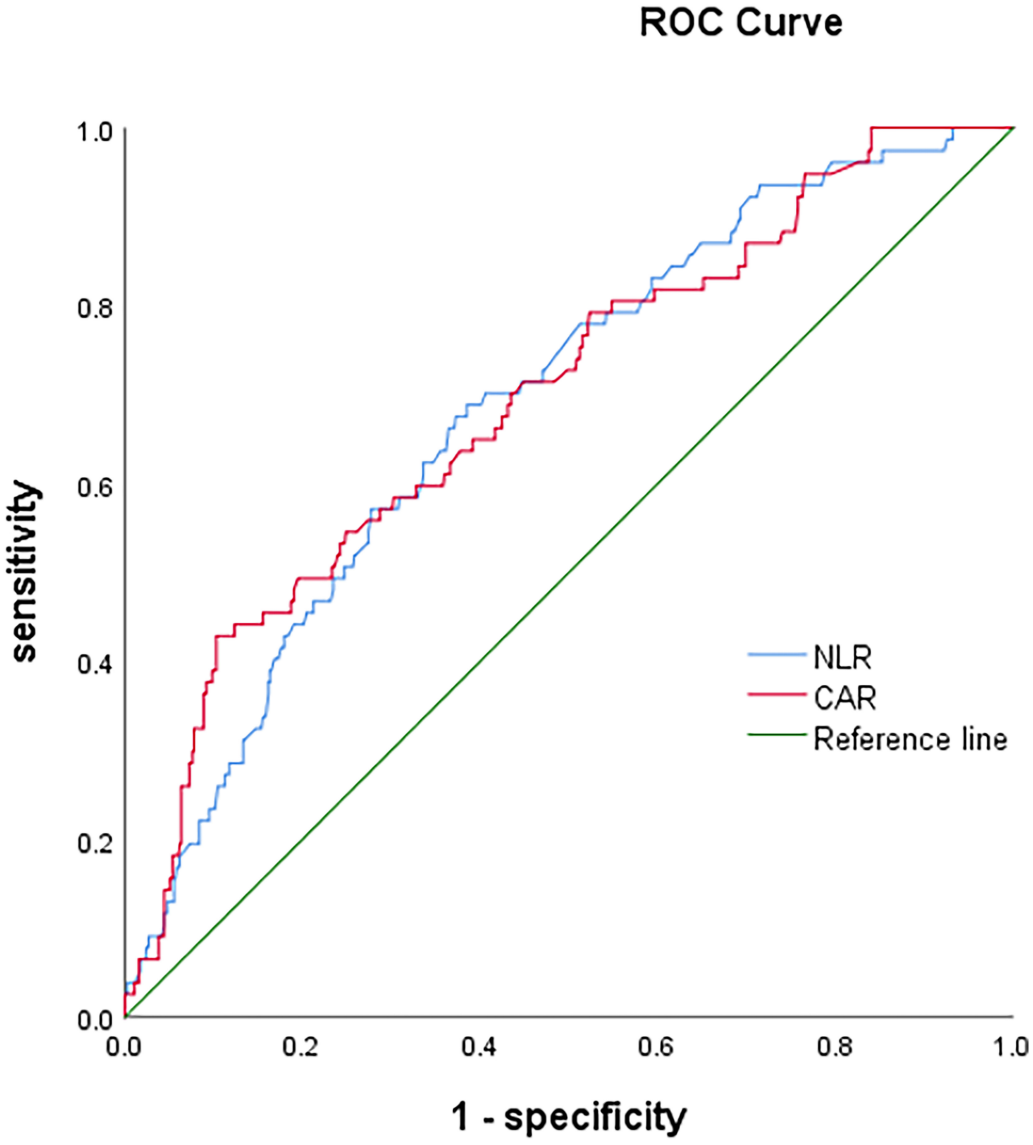
The receiver operating characteristic (ROC) curves of neutrophil-to-lymphocyte ratio (NLR) and c-reactive protein/albumin ratio (CAR).

Determined by the ROC curves, the optimal cut-off values of NLR and CAR were 2.2 and 0.07, and the corresponding areas under the curves (AUCs) were 0.688(95%CI: 0.628-0.748) and 0.696(95%CI: 0.633-0.759), respectively (**Table 2** and **Figure 2**). Patients were divided into low and high ratio groups based on cutoff values. 59.7%of the patients (423) had a low NLR (<2.2), and 40.3% (285) had a high NLR (≥2.2). 86.2% of patients (610) were categorized as low CAR (<0.07) and 13.8% (98) as high CAR (≥0.07).

**Table 2:** Receiver Operating Characteristics (ROC) Analyses of NLR and CAR in luminal breast cancers with HER2-negativity.

Variables	Cut-Off Value	AUC (95% CI)	Sensitivity	Specificity
NLR	2.2	0.688 (0.628-0.748)	0.675	0.628
CAR	0.07	0.696 (0.633-0.759)	0.429	0.902

### Associations Between Clinicopathological Characteristics and NLR or CAR Levels

Our analysis revealed that there were significant associations between NLR levels and Age(p=0.001), Menopausal status(p=0.01), Chemotherapy type(p=0.009). Similarly, CAR was strongly associated with Age(p=0.001), BMI(p=0.001), Menopausal status(p=0.001), Lymph node metastasis status(p=0.001), TNM grade(p=0.001), Histology type(p=0.006) and Operation type(p<0.001) (**Table 3**).

**Table 3:** Clinicopathological characteristics of breast cancers according to neutrophil-to-lymphocyte ratio (NLR) or c-reactive protein/albumin ratio (CAR) levels.

Characteristics	NLR		*p*-Value	CAR		*p*-Value
	NLR<2.2 (*n*=423)	NLR≥2.2 (*n*=285)		CAR<0.07 (n=610)	CAR≥0.07 (n=98)	
Age (years)			**0.001**			**0.001**
<50	185 (43.7%)	161 (56.5%)		316 (51.8%)	30 (30.6%)	
≥50	238 (56.3%)	124 (43.5%)		294 (48.2%)	68 (69.4%)	
BMI			0.305			**0.001**
<24	260 (61.5%)	186 (65.3%)		403 (66.1%)	43 (43.9%)	
≥24	163 (38.5%)	99 (34.7%)		207 (33.9%)	55 (56.1%)	
Menopausal status			**0.01**			**0.001**
Pre-	200 (48.4%)	162 (58.7%)		327 (55.1%)	35 (36.8%)	
Post-	213 (51.6%)	114 (41.3%)		267 (44.9%)	60 (63.2%)	
Tumor size			0.283			0.066
<2cm	275 (65.0%)	174 (61.1%)		395 (64.8%)	54 (55.1%)	
≥2cm	148 (35.0%)	111 (38.9%)		215 (35.2%)	44 (44.9%)	
Lymph node metastasis			0.108			**0.001**
Negative	370 (87.5%)	237 (83.2%)		535 (87.7%)	72 (73.5%)	
Positive	53 (12.5%)	48 (16.8%)		75 (12.3%)	26 (26.5%)	
TNM grade			0.093			**0.001**
I	257 (60.8%)	165 (57.9%)		377 (61.8%)	45 (45.9%)	
II	137 (32.4%)	87 (30.5%)		190 (31.1%)	34 (34.7%)	
III	29 (6.9%)	33 (11.6%)		43 (7.0%)	19 (19.4%)	
Histology type			0.444			**0.006**
Infiltrating ductal carcinoma	374 (88.6%)	261 (91.6%)		553 (90.7%)	82 (84.5%)	
Infiltrating lobular carcinoma	10 (2.4%)	5 (1.8%)		15 (2.5%)	0 (0)	
Others	38 (9.0%)	19 (6.7%)		42 (6.9%)	15 (15.5%)	
Luminal subtype			0.116			0.549
Luminal A	199 (47.0%)	117 (41.1%)		275 (45.1%)	41 (41.8%)	
Luminal B	224 (53.0%)	168 (58.9%)		335 (54.9%)	57 (58.2%)	
Operation type			0.216			**<0.001**
Modified radical mastectomy	225 (53.2%)	150 (52.6%)		314 (51.5%)	61 (62.2%)	
Breast-conserving surgery	147 (34.8%)	102 (35.8%)		226 (37.0%)	23 (23.5%)	
Mammectomy	47 (11.1%)	25 (8.8%)		64 (10.5%)	8 (8.2%)	
Others	4 (0.9%)	8 (2.8%)		6 (1.0%)	6 (6.1%)	
Chemotherapy			**0.009**			0.09
Neoadjuvant chemotherapy	32 (7.6%)	34 (11.9%)		51 (8.4%)	15 (15.3%)	
Adjuvant chemotherapy	206 (48.7%)	156 (54.7%)		315 (51.6%)	47 (48.0%)	
No chemotherapy	185 (43.7%)	95 (33.3%)		244 (40.0%)	36 (36.7%)	
Radiotherapy			0.175			0.811
No	194 (45.9%)	116 (40.7%)		266 (43.6%)	44 (44.9%)	
Yes	229 (54.1%)	169 (59.3%)		344 (56.4%)	54 (55.1%)	

All of our variables with p-values less than 0.05 are in bold.

### Prognostic Significance of the NLR and CAR

**Figure 3:**
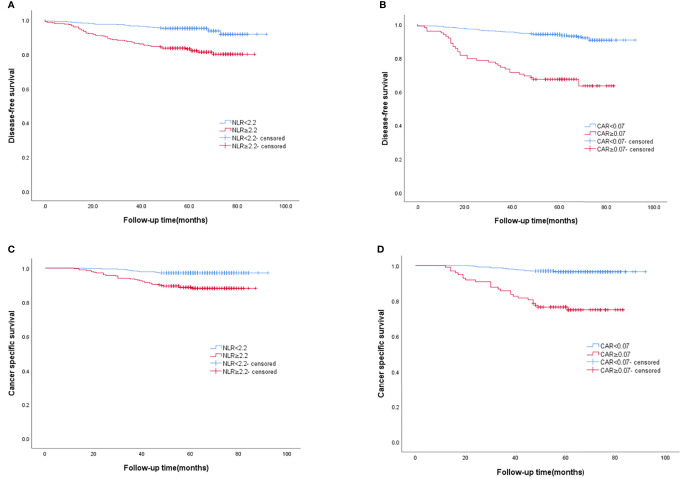
The Kaplan–Meier curves of DFS according to **(A)** NLR, **(B)** CAR, and the Kaplan–Meier curves of CSS according to **(C)** NLR, **(D)** CAR.

AS shown in **Figure 3**, the Kaplan–Meier curve was performed to analyze the DFS and CSS according to the cut-off values of NLR and CAR. The rate of DFS in BC patients was 89.1%, with a mean DFS of 60.76 (range 1–92) months. The DFS rate was lower in the group of NLR≥2.2 than that in the group of NLR<2.2 (81.8% vs. 94.1%, P<0.0001) (**Figure 3A**). Similar results were obtained among the group of CAR≥0.07 and the group of CAR<0.07(66.3% vs. 92.8%, P<0.0001) (**Figure 3B**). The rate of CSS in BC patients was 93.6%, with a mean CSS of 62.71 (range, 12-92) months. Similarly, longer CSS rate was recognized in the group of NLR<2.2 (97.2% vs. 88.4%, P<0.0001) (**Figure 3C**), and the group of CAR<0.07 (96.6% vs. 75.5%, P<0.0001) (**Figure 3D**).

### Univariable and Multivariable Analyses for DFS

By univariable analysis, Tumor size (P<0.001), Lymph node metastasis status (P<0.001), TNM stage III (P<0.001), Operation type (p=0.002), Chemotherapy type(P<0.001), NLR (P<0.001) and CAR (P<0.001) were significant predictors for DFS. In multivariate analysis that included these significant factors, NLR≥2.2 (HR=2.886, 95%CI=1.756-4.745, p<0.001) and CAR≥0.07 (HR=3.858, 95%CI=2.346-6.345, p<0.001) were significantly and independently associated with worse DFS. The risk of progression was increased in patients who received other types of operation (HR 0.458; P=0.005) than those who received modified radical mastectomy (**Table 4**).

**Table 4:** Univariable and multivariable analyses of DFS in Luminal BC with HER2-negativity.

Characteristics	*n*	Univariate analysisHR (95% CI)	*p*-Value	Multivariate analysisHR (95% CI)	*p*-Value
Age (years)			0.337		
<50	346	1			
≥50	362	1.246 (0.795-1.955)			
BMI			0.766		
<24	446	1			
≥24	262	1.072 (0.678-1.695)			
Menopausal status			0.669		
Pre-	362	1			
Post-	327	1.104 (0.702-1.736)			
Tumor size			**<0.001**		0.229
<2cm	449	1		1	
≥2cm	259	2.431 (1.548-3.818)		1.824 (0.685-4.856)	
Lymph node metastasis			**<0.001**		0.348
Negative	607	1		1	
Positive	101	4.567 (2.894-7.206)		1.567 (0.613-4.007)	
TNM grade					
I	422	1		1	
II	224	1.474 (0.843-2.576)	0.174	0.629 (0.200-1.981)	0.428
III	62	8.659 (5.098-14.706)	**<0.001**	1.740 (0.419-7.232)	0.446
Histology type					
Infiltrating ductal carcinoma	635	1	0.954		
Infiltrating lobular carcinoma	15	0	0.722		
Others	57	1.152 (0.530-2.505)			
Luminal subtype			0.054		
Luminal A	316	1			
Luminal B	392	1.591 (0.992-2.552)			
Operation type					
Modified radical mastectomy	375	1		1	
Breast-conserving surgery	249	0.393 (0.218-0.709)	**0.002**	0.651 (0.343-1.236)	0.189
Mammectomy	72	0.193 (0.047-0.794)	**0.023**	0.338 (0.079-1.440)	0.142
Others	12	9.573 (4.696-19.518)	**<0.001**	5.444 (2.253-13.155)	**<0.001**
Chemotherapy					
Neoadjuvant chemotherapy	66	1		1	
Adjuvant chemotherapy	362	0.304 (0.174-0.530)	**<0.001**	0.882 (0.444-1.751)	0.719
No chemotherapy	280	0.242 (0.131-0.447)	**<0.001**	0.894 (0.362-2.210)	0.809
Radiotherapy			0.506		
No	310	1			
Yes	398	0.859 (0.550-1.344)			
NLR			**<0.001**		**<0.001**
<2.2	423	1		1	
≥2.2	285	3.321 (2.061-5.352)		2.886 (1.756-4.745)	
CAR			**<0.001**		**<0.001**
<0.07	610	1		1	
≥0.07	98	5.624 (3.577-8.841)		3.858 (2.346-6.345)	

All of our variables with p-values less than 0.05 are in bold.

### Univariable and Multivariable Analyses for CSS

Tumor size(p=0.008), Lymph node metastasis status (p<0.001), TNM stage III (p<0.001), Luminal subtype(p=0.032), Operation type (p=0.015), Chemotherapy type(P<0.001), NLR (P<0.001) and CAR (P<0.001) were significant predictive factors for CSS by univariate analysis (Table II). Multivariate analysis was then conducted to evaluate all potential prognostic factors. The results indicated that the NLR≥2.2 (HR=3.999, 95%CI=2.002-7.987, p<0.001), CAR ≥0.07 (HR=6.563, 95%CI=3.558-12.106, p<0.001), Luminal B subtype (HR=2.912, 95%CI=1.464-5.794, p=0.002), other operation types (HR=2.964, 95%CI=1.055-8.331, p=0.039) were essentially and independently associated with worse CSS (**Table 5**).

**Table 5:** Univariable and multivariable analyses of CSS in Luminal BC with HER2-negativity.

Characteristics	n	Univariate analysisHR (95% CI)	p-Value	Multivariate analysisHR (95% CI)	p-Value
Age (years)			0.068		
<50	346	1			
≥50	362	1.765 (0.958-3.249)			
BMI			0.674		
<24	446	1			
≥24	262	1.137 (0.626-2.064)			
Menopausal status			0.2		
Pre-	362	1			
Post-	327	1.477 (0.813-2.682)			
Tumor size			**0.008**		0.774
<2cm	449	1		1	
≥2cm	259	2.222 (1.234-4.001)		1.185 (0.372-3.773)	
Lymph node metastasis			**<0.001**		0.846
Negative	607	1		1	
Positive	101	4.620 (2.557-8.349)		1.142 (0.301-4.337)	
TNM grade					
I	422	1		1	
II	224	1.297 (0.602-2.794)	0.507	1.136 (0.280-4.604)	0.858
III	62	8.634 (4.401-16.938)	**<0.001**	5.401 (0.864-33.769)	0.071
Histology type					
Infiltrating ductal carcinoma	635	1			
Infiltrating lobular carcinoma	15	0	0.965		
Others	57	1.101 (0.394-3.072)	0.855		
Luminal subtype			**0.032**		**0.002**
Luminal A	316	1		1	
Luminal B	392	2.026 (1.063-3.859)		2.912 (1.464-5.794)	
Operation type					
Modified radical mastectomy	375	1		1	
Breast-conserving surgery	249	0.358 (0.157-0.818)	**0.015**	0.596 (0.239-1.484)	0.266
Mammectomy	72	0.354 (0.084-1.482)	0.155	0.577 (0.126-2.628)	0.477
Others	12	10.833 (4.734-24.79)	**<0.001**	2.964 (1.055-8.331)	**0.039**
Chemotherapy					
Neoadjuvant chemotherapy	66	1		1	
Adjuvant chemotherapy	362	0.229 (0.108-0.484)	**<0.001**	0.727 (0.286-1.847)	0.503
No chemotherapy	280	0.318 (0.152-0.665)	**0.002**	1.902 (0.592-6.114)	0.280
Radiotherapy			0.829		
No	310	1			
Yes	398	1.067 (0.591-1.928)			
NLR			**<0.001**		**<0.001**
<2.2	423	1		1	
≥2.2	285	4.252 (2.196-8.233)		3.999 (2.002-7.987)	
CAR			**<0.001**		**<0.001**
<0.07	610	1		1	
≥0.07	98	8.066 (4.489-14.493)		6.563 (3.558-12.106)	

All of our variables with p-values less than 0.05 are in bold

## Discussion

Luminal BC with HER2-negativity is the most common subtype of BC and has the best prognosis compared to other subtypes (23). However, studies on long-term survival have shown that a proportion of HER2-negative luminal BC patients can still relapse and metastases, resulting in poor prognosis. Thus, reliable prognostic factors are needed for further classification in order to personalize treatment and follow-up. Current methods used to predict the prognosis of patients with BC include determination of the tumor node metastasis (TNM) stage, Ki67 expression, receptor status and genetic testing (24–26). However, these prognosis-related tests can be invasive and cost time and money. By contrast, blood-based tests for inflammatory parameters are simple, maneuverable, cheap and easily promoted.

At present, it has become a consensus that inflammatory response carries a key role in the pathophysiology of tumor. Recently, substantial evidence have shown that inflammatory biomarkers are considered to correlate with the prognosis of various malignancies, including BC (6–9). NLR, one of the most frequently applied indicators, has been confirmed to be an independent prognostic indicator in BC, but its significance as a prognostic factor for each BC subtype remains unclear (15). In addition, CAR, a novel index related with inflammation and nutrition, has been demonstrated as a predictor for the prognosis of several cancers (17–20), yet previous studies did not correlate CAR with the prognosis of luminal BC with HER2-negativity. Given the fact that few studies investigated the value of NLR and CAR as prognostic markers for luminal BC with HER2-negativity, we attempted to investigate the predictive value of NLR and CAR on DFS and CSS for luminal BC patients with HER2-negativity by univariate and multivariate analyses.

Our research revealed that NLR and CAR were independent prognostic factors for luminal breast cancers with HER2-negativity. Univariate and multivariate analysis confirmed that lower NLR (<2.2) was significantly associated with favorable DFS (P<0.001), as well as CSS (P<0.001). Similarly, lower CAR (<0.07) was independently and significantly associated with longer DFS (P<0.001) and CSS (P<0.001). Our results were consisted with the previous studies. Bun et al. (15) retrospectively studied 677 operated BC patients and identified that the prognostic significance of NLR is limited to ER-positive/HER2-negative breast cancers, low NLR was an independent predictor for better prognosis in this subgroup (p=0.022). Noh et al. (27) reported that high NLR (≥2.5) predicted lower disease-specific survival in 442 BC patients and luminal A subtype was the only intrinsic subtype in which patients with higher NLR showed significantly poor prognosis (87.7% vs. 96.7%, p=0.009). Koh et al. (16) evaluated the predictive value of NLR in a retrospective study among 157 ER/PR positive and HER-2 negative BC patients who have received neoadjuvant chemotherapy. In this study, univariate and multivariate Cox analysis showed that NLR was an independent prognostic factor for RFS (P=0.001) and OS (P<0.001). CAR was developed as a prognostic score for patients with sepsis and was later widely used for several cancers, including esophageal cancer (17), gastric cancer (18), pancreatic cancer (19) and ovarian cancer (20). However, there was few studies focusing on the prognostic role of CAR in BC. Zhou et al. (9) reported that an increased preoperative CAR (≥0.029) was an independent risk factor for decreased OS (HR, 9.189; P=0.003) and DFS (HR, 2.225; P=0.024) in patients with non-metastatic BC. By contrast, Wang et al. (21) identified that there was no association between elevated CAR (≥0.34) and the survival of BC patients with skeletal metastases in multivariate analysis. There are several explanations for these contradicting results such as differences in the optimal cut-off value for CAR, the selection of study subjects, the timing of the pre-treatment blood samples and the follow-up time.

A high neutrophil level has been implicated to play a pivotal role in promoting the development of tumor. Neutrophils can release a series of proteins or chemokines, such as Neutrophil Elastase (NE), myeloperoxidase (MPO), cytokines, fibroblast growth factor-2 (FGF-2), matrix metalloproteinase-9 and VEGF to promote tumor proliferation, invasion and angiogenesis (28–30). In addition, neutrophils can produce argininase-1 and hydrogen peroxide to inhibit the lytic activity of activated T cells, natural killer cells, and lymphocytes, thereby causing disorder in the immune system (31, 32). A recent basic study suggested that neutrophils can cluster around circulating tumor cells (CTCs), induce tumor cell aggregation, and help tumor cells survive by hiding them from immune surveillances (33). Lymphocytes are the main components involved in tumor immune response and serve as an anti-tumor role either by directly killing tumor cells through their lymphocyte-mediated cytotoxicity, or by activating the immune system to release multiple cytokines (34). Besides, low lymphocyte counts are thought to be associated with inadequate immune response, which may increase the risk of tumor recurrence or metastasis (35). Therefore, elevated NLR (increased neutrophils and/or decreased lymphocytes) might be related to immunodeficiency in luminal BC patients with HER2-negativity, which may partly explain why NLR can be used as a prognostic indicator in BC.

CRP is well-known as an acute phase inflammatory marker, which is synthesized by hepatocytes and induced by pro-inflammatory cytokines. Increased levels of CRP in serum have been reported to be associated with invasion, metastasis and poor prognosis of BC (36, 37). CRP can promote tumor cell invasion and metastasis through the activation of integrin α2 signaling in the inflammatory microenvironment (38). Moreover, angiogenesis can also be accelerated by CRP *via* increasing the levels of vascular growth factors and interleukins (39). Albumin is a circulating protein in plasma that reflects the nutritional status of patients. Tumor depletion and inflammation often lead to decreased serum albumin levels in cancer patients, while malnutrition can suppress immune defense mechanisms and contribute to tumor progression (40). Additionally, several studies also have shown that low preoperative albumin level is an independent predictor for poor prognosis in a variety of malignancies, including BC (41, 42). Since CAR can reflect the inflammation and nutritional status of cancer patients in a more comprehensive way, the serum marker that was originally used to assess the prognosis of patients with sepsis, was later introduced to prognosing various malignancies (17–20).

According to our study, luminal HER2-negative BC patients with an increased NLR or CAR were more likely to have a poorer prognosis. For these patients, more frequent follow-up should be performed and more powerful adjuvant therapy could be administered to prevent recurrence and prolong survival postoperatively.

However, our study still had several limitations. This is a single-center retrospective study with inevitable bias, and the specific mechanisms of NLR and CAR in BC remain unclear. Besides, we set the cut-off values of NLR and CAR at 2.2 and 0.07 respectively by ROC curve based on our data. Whether they are the best optimal cut-off values remains unclear. In this regard, whether these cut-off values can be widely applied in clinical practice still needs further investigations. In addition, only luminal HER2-negative BC patients were studied in this paper, and other types of breast cancer may not be applicable to this conclusion. Therefore, more prospective, large-scale and multi-center clinical studies are needed in the future to further clarify the relationship between NLR and CAR and the prognosis of BC and the mechanism, so as to identify and screen high-risk groups and provide targeted interventions timely.

## Conclusion

In conclusion, our results suggested that elevated NLR and CAR prior to treatment were both significantly and independently correlated with poorer DFS and CSS in luminal breast cancers with HER2-negativity. Given their economical, convenient and highly operative features, NLR and CAR are promising to be used as an effective method of identifying patients with poor prognosis in daily clinical practice and help to provide a more accurate treatment timely. In addition, whether anti-inflammatory therapy can improve the sensitivity to chemotherapy drugs and the prognosis among BC patients still need to be further explored in the future.

## Data Availability Statement

The original contributions presented in the study are included in the article/supplementary material. Further inquiries can be directed to the corresponding authors.

## Ethics Statement

The studies involving human participants were reviewed and approved by Sir Run Run Shaw Hospital Affiliated to Zhejiang University School of Medicine. Written informed consent for participation was not required for this study in accordance with the national legislation and the institutional requirements.

## Author Contributions

FC designed the work and wrote the manuscript. DC and LJ analyzed and interpreted the patient data. CX was responsible for the management and coordination of the planning and execution of research activities. WZ and WH revised the manuscript. FC was the major contributor in writing the manuscript. All authors contributed to the article and approved the submitted version.

## Funding

This study was supported by the National Natural Science Foundation of China (No. 81874142) and Zhejiang Provincial Natural Science Foundation of China (No. LY22H160011, No. LXR22H160001).

## Conflict of Interest

The authors declare that the research was conducted in the absence of any commercial or financial relationships that could be construed as a potential conflict of interest.

## Publisher’s Note

All claims expressed in this article are solely those of the authors and do not necessarily represent those of their affiliated organizations, or those of the publisher, the editors and the reviewers. Any product that may be evaluated in this article, or claim that may be made by its manufacturer, is not guaranteed or endorsed by the publisher.

## References

[1] Siegel RL Miller KD Jemal A. Cancer Statistics, 2020. CA Cancer J Clin (2020) 70(1):7–30. doi: 10.3322/caac.21590 31912902

[2] Bray F Ferlay J Soerjomataram I Siegel RL Torre LA Jemal A. Global Cancer Statistics 2018: GLOBOCAN Estimates of Incidence and Mortality Worldwide for 36 Cancers in 185 Countries. CA Cancer J Clin (2018) 68(6):394–424. doi: 10.3322/caac.21492 30207593

[3] Chen W Zheng R Baade PD Zhang S Zeng H Bray F. Cancer Statistics in China, 2015. CA Cancer J Clin (2016) 66(2):115–32. doi: 10.3322/caac.21338 26808342

[4] Chen X Xu C Hong S Xia X Cao Y McDermott J. Immune Cell Types and Secreted Factors Contributing to Inflammation-To-Cancer Transition and Immune Therapy Response. Cell Rep (2019) 26(7):1965–77.e4. doi: 10.1016/j.celrep.2019.01.080 30759403

[5] Fridman WH Zitvogel L Sautès-Fridman C Kroemer G. The Immune Contexture in Cancer Prognosis and Treatment. Nat Rev Clin Oncol (2017) 14(12):717–34. doi: 10.1038/nrclinonc.2017.101 28741618

[6] Cupp MA Cariolou M Tzoulaki I Aune D Evangelou E Berlanga-Taylor AJ. Neutrophil to Lymphocyte Ratio and Cancer Prognosis: An Umbrella Review of Systematic Reviews and Meta-Analyses of Observational Studies. BMC Med (2020) 18(1):360. doi: 10.1186/s12916-020-01817-1 33213430 PMC7678319

[7] Lin S Fang Y Mo Z Lin Y Ji C Jian Z. Prognostic Value of Lymphocyte to Monocyte Ratio in Pancreatic Cancer: A Systematic Review and Meta-Analysis Including 3338 Patients. World J Surg Oncol (2020) 18(1):186. doi: 10.1186/s12957-020-01962-0 32711514 PMC7382838

[8] Li B Zhou P Liu Y Wei H Yang X Chen T. Platelet-To-Lymphocyte Ratio in Advanced Cancer: Review and Meta-Analysis. Clin Chim Acta (2018) 483:48–56. doi: 10.1016/j.cca.2018.04.023 29678631

[9] Zhou L Ma S Balde AI Han S Cai Z Li Z. A Retrospective Propensity Score Matched Study of the Preoperative C-Reactive Protein to Albumin Ratio and Prognosis in Patients With Resectable Non-Metastatic Breast Cancer. Med Sci Monit (2019) 25:4342–52. doi: 10.12659/msm.913684 PMC6582690 31182704

[10] Wang D Duan L Tu Z Yan F Zhang C Li X. The Glasgow Prognostic Score Predicts Response to Chemotherapy in Patients With Metastatic Breast Cancer. Chemotherapy (2016) 61(4):217–22. doi: 10.1159/000443367 26905743

[11] Ethier JL Desautels D Templeton A Shah PS Amir E. Prognostic Role of Neutrophil-to-Lymphocyte Ratio in Breast Cancer: A Systematic Review and Meta-Analysis. Breast Cancer Res (2017) 19(1):2. doi: 10.1186/s13058-016-0794-1 28057046 PMC5217326

[12] Guo W Lu X Liu Q Zhang T Li P Qiao W. Prognostic Value of Neutrophil-to-Lymphocyte Ratio and Platelet-to-Lymphocyte Ratio for Breast Cancer Patients: An Updated Meta-Analysis of 17079 Individuals. Cancer Med (2019) 8(9):4135–48. doi: 10.1002/cam4.2281 PMC6675722 31197958

[13] Tiainen S Rilla K Hamalainen K Oikari S Auvinen P. The Prognostic and Predictive Role of the Neutrophil-to-Lymphocyte Ratio and the Monocyte-to-Lymphocyte Ratio in Early Breast Cancer, Especially in the HER2+ Subtype. Breast Cancer Res Treat (2020) 185(1):63–72. doi: 10.1007/s10549-020-05925-7 32948994 PMC7500503

[14] Ding N Huang J Li N Yuan J Wang S Xiao Z. Roles of Neutrophil/Lymphocyte Ratio in Prognosis and in Differentiation of Potential Beneficiaries in HER2-Positive Breast Cancer With Trastuzumab Therapy. BMC Cancer (2020) 20(1):235. doi: 10.1186/s12885-020-06750-3 32192443 PMC7082930

[15] Bun A Fujimoto Y Higuchi T Sata A Fukui R Ozawa H. Prognostic Significance of Neutrophil-to-Lymphocyte Ratio in Luminal Breast Cancers With Low Levels of Tumour-Infiltrating Lymphocytes. Anticancer Res (2020) 40(5):2871–80. doi: 10.21873/anticanres.14263 32366437

[16] Koh YW Lee HJ Ahn JH Lee JW Gong G. Prognostic Significance of the Ratio of Absolute Neutrophil to Lymphocyte Counts for Breast Cancer Patients With ER/PR-Positivity and HER2-Negativity in Neoadjuvant Setting. Tumour Biol (2014) 35(10):9823–30. doi: 10.1007/s13277-014-2282-5 24986572

[17] Wang Y Hu X Huang Y Xu WY Wu YM Li PF. Prognostic Value of the C-Reactive Protein to Albumin Ratio in Esophageal Cancer: A Systematic Review and Meta-Analysis. Kaohsiung J Med Sci (2020) 36(1):54–61. doi: 10.1002/kjm2.12129 31512813 PMC11896523

[18] Yang X Song X Zhang L Wu C. Prognostic Role of the Pretreatment C-Reactive Protein/Albumin Ratio in Gastric Cancer: A Systematic Review and Meta-Analysis. Med (Baltimore) (2020) 99(10):e19362. doi: 10.1097/md.0000000000019362 PMC7478778 32150079

[19] Zang Y Fan Y Gao Z. Pretreatment C-Reactive Protein/Albumin Ratio for Predicting Overall Survival in Pancreatic Cancer: A Meta-Analysis. Med (Baltimore) (2020) 99(23):e20595. doi: 10.1097/md.0000000000020595 PMC7306286 32502031

[20] Komura N Mabuchi S Shimura K Kawano M Matsumoto Y Kimura T. Significance of Pretreatment C-Reactive Protein, Albumin, and C-Reactive Protein to Albumin Ratio in Predicting Poor Prognosis in Epithelial Ovarian Cancer Patients. Nutr Cancer (2021) 73(8):1357–64. doi: 10.1080/01635581.2020.1798479 32835520

[21] Wang Y Huang G Li Z. Prognostic Significance of Inflammatory Biomarkers in Patients With Breast Cancer Skeletal Metastases. Cancer Manag Res (2020) 12:11463–75. doi: 10.2147/cmar.s277291 PMC7665573 33204159

[22] Goldhirsch A Wood WC Coates AS Gelber RD Thürlimann B Senn HJ. Strategies for Subtypes–Dealing With the Diversity of Breast Cancer: Highlights of the St. Gallen International Expert Consensus on the Primary Therapy of Early Breast Cancer 2011. Ann Oncol (2011) 22(8):1736–47. doi: 10.1093/annonc/mdr304 PMC3144634 21709140

[23] Hennigs A Riedel F Gondos A Sinn P Schirmacher P Marmé F. Prognosis of Breast Cancer Molecular Subtypes in Routine Clinical Care: A Large Prospective Cohort Study. BMC Cancer (2016) 16(1):734. doi: 10.1186/s12885-016-2766-3 27634735 PMC5024419

[24] Lyman GH Somerfield MR Bosserman LD Perkins CL Weaver DL Giuliano AE. Sentinel Lymph Node Biopsy for Patients With Early-Stage Breast Cancer: American Society of Clinical Oncology Clinical Practice Guideline Update. J Clin Oncol (2017) 35(5):561–4. doi: 10.1200/jco.2016.71.0947 27937089

[25] Hadad SM Jordan LB Roy PG Purdie CA Iwamoto T Pusztai L. A Prospective Comparison of ER, PR, Ki67 and Gene Expression in Paired Sequential Core Biopsies of Primary, Untreated Breast Cancer. BMC Cancer (2016) 16(1):745. doi: 10.1186/s12885-016-2788-x 27658825 PMC5034430

[26] Zheng H Luo L Zhao W. Factors Associated With Level III Lymph Nodes Positive and Survival Analysis of its Dissection in Patients With Breast Cancer. Laparosc Endosc Robot Surg (2020) 3(2):43–7. doi: 10.1016/j.lers.2020.03.001

[27] Noh H Eomm M Han A. Usefulness of Pretreatment Neutrophil to Lymphocyte Ratio in Predicting Disease-Specific Survival in Breast Cancer Patients. J Breast Cancer (2013) 16(1):55–9. doi: 10.4048/jbc.2013.16.1.55 PMC3625770 23593082

[28] Liang W Ferrara N. The Complex Role of Neutrophils in Tumor Angiogenesis and Metastasis. Cancer Immunol Res (2016) 4(2):83–91. doi: 10.1158/2326-6066.cir-15-0313 26839309

[29] Li Z Takino T Endo Y Sato H. Activation of MMP-9 by Membrane Type-1 MMP/MMP-2 Axis Stimulates Tumor Metastasis. Cancer Sci (2017) 108(3):347–53. doi: 10.1111/cas.13134 PMC5378257 27987367

[30] Coffelt SB Wellenstein MD de Visser KE. Neutrophils in Cancer: Neutral No More. Nat Rev Cancer (2016) 16(7):431–46. doi: 10.1038/nrc.2016.52 27282249

[31] Schmielau J Finn OJ. Activated Granulocytes and Granulocyte-Derived Hydrogen Peroxide Are the Underlying Mechanism of Suppression of T-Cell Function in Advanced Cancer Patients. Cancer Res (2001) 61(12):4756–60. 11406548

[32] Bronte V Zanovello P. Regulation of Immune Responses by L-Arginine Metabolism. Nat Rev Immunol (2005) 5(8):641–54. doi: 10.1038/nri1668 16056256

[33] Hurt B Schulick R Edil B El Kasmi KC Barnett C Jr. Cancer-Promoting Mechanisms of Tumor-Associated Neutrophils. Am J Surg (2017) 214(5):938–44. doi: 10.1016/j.amjsurg.2017.08.003 28830617

[34] Fridman WH Pagès F Sautès-Fridman C Galon J. The Immune Contexture in Human Tumours: Impact on Clinical Outcome. Nat Rev Cancer (2012) 12(4):298–306. doi: 10.1038/nrc3245 22419253

[35] Zhu J Powis de Tenbossche CG Cané S Colau D van Baren N Lurquin C. Resistance to Cancer Immunotherapy Mediated by Apoptosis of Tumor-Infiltrating Lymphocytes. Nat Commun (2017) 8(1):1404. doi: 10.1038/s41467-017-00784-1 29123081 PMC5680273

[36] Petekkaya I Unlu O Roach EC Gecmez G Okoh AK Babacan T. Prognostic Role of Inflammatory Biomarkers in Metastatic Breast Cancer. J Buon (2017) 22(3):614–22. 28730765

[37] Guo L Liu S Zhang S Chen Q Zhang M Quan P. C-Reactive Protein and Risk of Breast Cancer: A Systematic Review and Meta-Analysis. Sci Rep (2015) 5:10508. doi: 10.1038/srep10508 26001129 PMC5377048

[38] Kim ES Kim SY Koh M Lee HM Kim K Jung J. C-Reactive Protein Binds to Integrin α2 and Fcγ Receptor I, Leading to Breast Cell Adhesion and Breast Cancer Progression. Oncogene (2018) 37(1):28–38. doi: 10.1038/onc.2017.298 28846105

[39] Kim ES Cha Y Ham M Jung J Kim SG Hwang S. Inflammatory Lipid Sphingosine-1-Phosphate Upregulates C-Reactive Protein *via* C/Ebpβ and Potentiates Breast Cancer Progression. Oncogene (2014) 33(27):3583–93. doi: 10.1038/onc.2013.319 23955082

[40] Mantzorou M Koutelidakis A Theocharis S Giaginis C. Clinical Value of Nutritional Status in Cancer: What is Its Impact and How it Affects Disease Progression and Prognosis? Nutr Cancer (2017) 69(8):1151–76. doi: 10.1080/01635581.2017.1367947 29083236

[41] Gupta D Lis CG. Pretreatment Serum Albumin as a Predictor of Cancer Survival: A Systematic Review of the Epidemiological Literature. Nutr J (2010) 9:69. doi: 10.1186/1475-2891-9-69 21176210 PMC3019132

[42] Oñate-Ocaña LF Aiello-Crocifoglio V Gallardo-Rincón D Herrera-Goepfert R Brom-Valladares R Carrillo JF. Serum Albumin as a Significant Prognostic Factor for Patients With Gastric Carcinoma. Ann Surg Oncol (2007) 14(2):381–9. doi: 10.1245/s10434-006-9093-x 17160496

